# Investigation of boiler energy consumption in the gas refinery units using RSM ANN and Aspen HYSYS

**DOI:** 10.1016/j.heliyon.2024.e41450

**Published:** 2024-12-24

**Authors:** Erfan Gholamzadeh, Ahad Ghaemi, Abolfazl Shokri, Bahman Heydari

**Affiliations:** a School of Chemical, Petroleum and Gas Engineering, Iran University of Science and Technology, Tehran, Iran; b Faculty of Mechanical Engineering, University of Tehran, Iran

**Keywords:** Efficiency, Boiler, Optimization, ANN, RSM, Aspen HYSYS

## Abstract

In order to lower total energy consumption, this study focuses on optimizing energy use in refinery boilers. Using Aspen HYSYS simulations and modeling approaches like Artificial Neural Networks (ANNs) and Response Surface Methodology (RSM), data from 579 days of boiler operation was gathered and examined. Radial Basis Function (RBF) and Multi-Layer Perceptron (MLP) techniques were used in the ANN modeling. Under the same operating circumstances, Aspen HYSYS estimated an energy usage of 1,355 m³, whereas the actual consumption was 986 m³. While the R^2^ values for the ANN models were 0.98 for the RBF model and 0.99 for the MLP model, the R^2^ value derived using RSM was 0.97. Furthermore, the RBF model's performance metrics were 0.0034, whereas the MLP model's were 0.0018. The MLP model is the best choice, according to these findings. It is estimated that burning 26,000 m³ of fuel with an air supply of 23 m³/h at 25.5 °C will result in a steam flow of 525.5 tons per day at 10.5 barg and 256.5 °C. According to actual statistics, these circumstances might prevent the release of 27 tons of carbon dioxide by reducing fuel usage by over 10,000 m³ per hour. By optimizing the combustion stack's air supply, this decrease is accomplished.

## 1 Introduction

Chemical industries consume significant energy resources for various operations, including process equipment functionality and heating requirements. Current energy sources include fossil fuels, wind, solar, geothermal, nuclear, and hydropower, with fossil fuels being the most widely used. In chemical industries, energy carriers predominantly include electricity and steam. The most effective use of fossil fuels involves combustion, which releases internal energy as heat, transferred to the environment as radiation. Boilers and gas turbines are commonly used to generate steam and electricity through fossil fuel combustion. Research indicates that improving industrial process efficiency and energy conversion devices, as well as implementing energy management programs, are key strategies for rationalizing energy consumption globally [1].

Numerous studies have explored boiler energy consumption, aiming to enhance the efficiency of this critical equipment or review various efficiency-improving projects. Shi et al. investigated the performance of a supercritical boiler in a coal power plant, using CFD data alongside historical data for ANN training [2]. Smrekar et al. examined two ANN models, finding both to be suitable based on mean square error and correlation coefficients [3]. However, actual management of these systems involves operators evaluating energy performance, detecting abnormal consumption, and identifying improvements. Recent advancements in data analysis, such as artificial intelligence (AI), assist operators in analyzing equipment data and making informed decisions to enhance energy performance [4].

Sohrabi Kashani initially analyzed combustion performance in power plant boilers through energy and material balance calculations and real-world combustion conditions. The study highlighted energy losses due to unoptimized air-fuel ratios and proposed solutions to reduce stack energy losses by determining optimal combustion conditions [5]. Kochaki et al. used energy and exergy analysis to improve boiler performance in the fourth refinery of the South Pars gas complex. They investigated environmental factors affecting boiler efficiency, including ambient temperature, air humidity, excess air percentage, and water preheating. Their results showed that adjusting the excess air percentage could improve exergy efficiency to 41.46 % [6]. Mojica Cabeza compared mathematical methods for calculating boiler energy efficiency, concluding that empirical models are more practical for industrial applications when a comprehensive database is available. In energy data analysis, detecting abnormal consumption behaviors is crucial [7]. Various methods exist for identifying anomalies, which may result from device malfunctions, user negligence, theft, or non-technical damage. AI has proven effective in detecting such anomalies and understanding their causes [8].

The primary goal of this research is to identify operating points where the boiler exhibits the lowest energy consumption while producing steam within a fixed range, utilizing RSM, ANN, and Aspen HYSYS. The study involves analyzing energy consumption data and performance factors of refinery boilers, creating performance diagrams, and determining the optimal operating point. This research aims to evaluate different boiler conditions based on steam production requirements, selecting the best conditions with RSM, ANN, and Aspen HYSYS, and verifying the results with actual data to identify potential fuel consumption optimizations.

### 1.1 Calculating energy use in refineries

**Fig. 1:**
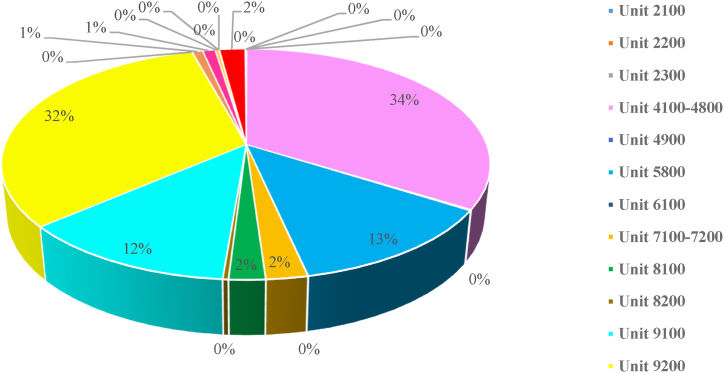
The percentage of energy consumed by refinery process equipment.

Assessing the energy consumption of a gas refinery reveals that the boiler is a significant energy consumer. Evaluating its energy performance and implementing enhancement projects can result in substantial energy savings. To facilitate this, the energy consumption of both the main and secondary units of the refinery is first measured and recorded. Fig. 1 illustrates the energy consumption of various refinery units and their contribution to the total energy consumption.

Fig. 1 highlights that the gas refinery's highest energy consumption is attributed to the sweetening units and the steam production and distribution units, accounting for 34 % and 32 %, respectively. Further analysis of the gas refinery equipment is shown in the following diagram.

**Fig. 2:**
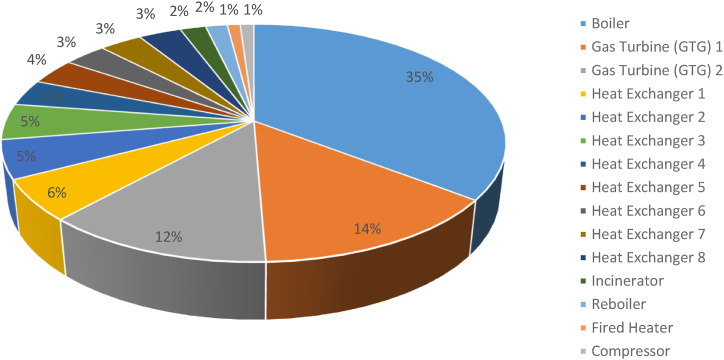
The percentage of energy consumed by refinery process units.

**Fig. 3:**
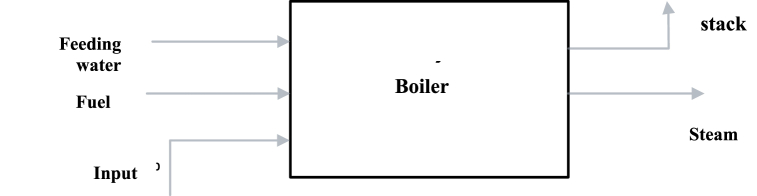
Boiler inlets and outlets.

Fig. 2 indicates that 32 % of the refinery's total energy consumption is attributable to boilers, which significantly surpasses the 12 % consumption by gas turbines. Therefore, boilers are critical, and optimizing their fuel consumption should be a priority. The boiler features three inlets and two outlets (Fig. 3), where the inlets include water, air entering the combustion stack, and fuel needed for combustion, while the outlets comprise exhaust gases and produced steam. These elements represent the energy and material flows entering or exiting the system boundaries and the equipment's energy consumption or production.

The refinery in question requires approximately 375,000 kg/h of steam under normal conditions and up to 434,000 kg/h during peak consumption, supplied by 11 boilers. The boiler under study has a capacity of 32–47 tons/h. The water entering the boiler has a temperature of 110 °C and a pressure of 13 barg, while the steam produced has a pressure between 10 and 11.5 barg and a temperature between 260 and 270 °C. The boiler's fuel is supplied from the LPG production unit, consisting of propane and butane at 11 barg pressure. Daily input and output data for the boiler are recorded by the operator, with data collected for up to 579 days, and summarized in Table 1.

**Table 1:** Minimum and maximum data collected.

Boiler	Flow Steam ton/d	P Steam barg	T Steam °C	Flow Air m^3^/hr	T Air °C	T Stack °C	SG of fuel	LHV of fuel	Energy
min	83	9.4	232.0	8.2	8.0	153.3	0.6	44640	12400
max	957	11.0	273.0	377.7	134.5	541.8	0.7	46910	64530

To analyze the boiler's energy performance, all influencing factors are identified and categorized into static factors (if any) and relevant variables. Static factors are consistent and do not change routinely, whereas relevant variables vary regularly and should be measurable and quantifiable. Ensuring the precision and accuracy of these measurements is crucial. Energy consumption data and relevant variables affecting performance are recorded at specific intervals. To evaluate the boiler's energy performance, factors such as gas consumption, output steam flow rate, output steam pressure, output steam temperature, input air flow rate to the combustion stack, ambient air temperature, and exhaust gas temperature are analyzed.

The steam flow rate is recorded using a steam flowmeter located in the boiler header, while pressure and temperature are measured with gauges installed in the boiler header. The air flow rate is measured using an air flowmeter in the air inlet channel, and the stack thermometer measures the exhaust gas temperature. Increased steam output leads to higher water input and fuel consumption. Similarly, higher steam temperature and pressure result in greater fuel consumption. Optimizing the temperature of the water entering the boiler can reduce fuel consumption. Proper combustion requires an optimal balance of air (oxygen), fuel, and heat. Increased air and fuel temperatures result in reduced fuel consumption. Monitoring the temperature of exhaust gases can help ensure efficient combustion, as lower exhaust gas temperatures indicate better combustion and lower fuel consumption. By adjusting these parameters and identifying the optimal operating point, the boiler's efficiency can be maximized. This study uses Response Surface Methodology (RSM), Artificial Neural Networks (ANNs), and Aspen HYSYS techniques to determine the most efficient operating point.

## 2 Modeling and simulation

### 2.1 Aspen HYSYS

Representing chemical or physical changes using a mathematical model that computes mass and energy balances, phase equilibria, and chemical transport and kinetic equations is the aim of chemical process simulation. This process aims to predict the behavior of a known process structure, given the basic data for the equipment involved.

Linear, nonlinear, and differential algebraic equations that represent equipment operations, physical-chemical qualities, equipment connections, and operational characteristics are all included in the mathematical model used in process simulation. The process flow diagram, which is the common language for chemical processes, illustrates these interactions. Process flow diagrams show the current condition of both hypothetical and real-world processes. The data in these diagrams is then interpreted and analyzed by process simulators to evaluate process performance and forecast any breakdowns.

Equations of state are models used to fit pressure-volume-temperature (PVT) correlations, facilitating the estimation of properties for pure substances or mixtures through Maxwell's relations. For mixtures, mixing rules are employed to achieve accurate estimations. Cubic equations of state are particularly significant due to their mathematical simplicity and effective approximation of theoretical property values. In this simulation, the NRTL (Non-Random Two-Liquid) model is used, developed by Renon and Prausnitz in 1968. This model extends the local composition concept to account for non-random interactions between molecules.

### 2.2 RSM modeling

RSM is a useful technique for assessing, simulating, and examining the impact of various operational variables' outcomes as well as their relationships. The empirical models produced the fitting datasets associated with the experimental testing, and the RSM incorporates the resulting statistical techniques and mathematical linkages [9]. Many scholars have been interested in the response surface approach in recent years. This method analyzes the interaction relationships between operational factors using a set of mathematical and statistical-based techniques, even if numerous input variables have an impact on response characteristics and process performance [10]. This method's system and replies are able to optimize the answer by taking into account conditions. A process's performance must be modeled using at least one quadratic model and one higher-order quadratic or nonlinear model in order to completely characterize it and potential changes in operating circumstances. Eq. (1) represents the quadratic model's regression [11].

(1) $$y=a_{0}+\sum_{i=1}^{n}a_{i}{\;x}_{i}\sum_{i=1}^{n}a_{ii}\;x_{i}^{2}\sum_{i}^{n}\;\sum_{j}^{n}a_{ii}{\;x}_{i}{\;x}_{j}+\varepsilon$$

where the process response is denoted by y, the model error is denoted by ε, the process variables T and P are denoted by X_i_ and X_j_, the first-order effect coefficients by αi and α_j_, the interaction coefficients by α_ii_ and α_ij_, and the parameter number by n. Utilize the method of least squares to get the least amount of residual from the coefficient b [12].

## 3 Neural network model

The neural network approach, which corresponds to the configuration of the human brain and nerves, has become increasingly popular in recent decades. The neural network is a statistical and computational model that uses the patterns of biological neural networks to simulate phenomena [13]. High processing speed for solving complex problems and the relationship between input and output data, as well as network adaptability, response to error data, error tolerance, and learning, are all advantages of the neural networks. Neurons are the smallest units of data analysis. Numbers are used as input and output data in the neuron unit. A cell's feedback signals are added together. Finding suitable weight values (w) for a given function (f) is the aim of an ANN. Following the addition of each input (x_i_) and its matching input weight (w_i_) to the bias (b), the output threshold value, or y, is shown in Eq. (2) [9].

(2) $$net=\left(\sum_{i=l}^{n}\omega i\;xi\right)+b$$

The results in the transfer function (f), and Eq. (3) produce the output values (y).

(3) y=f(net)

Ramp, linear, step, and sigmoid are the most often used transfer functions. In an ANN, learning involves learning algorithms to estimate the coordination weights of neurons. One of the most popular neural networks for producing non-linear maps is the MLP. Eq. (4) is the foundation of the MLP feature technique. The output vector is denoted by g, the reference vector and weighted vector of coefficients by x_k_, and the threshold limit is denoted by θ [14].

(4) $$g=f\left(w\;{xi}^{k}+\theta \;\right)$$

**Fig. 4:**
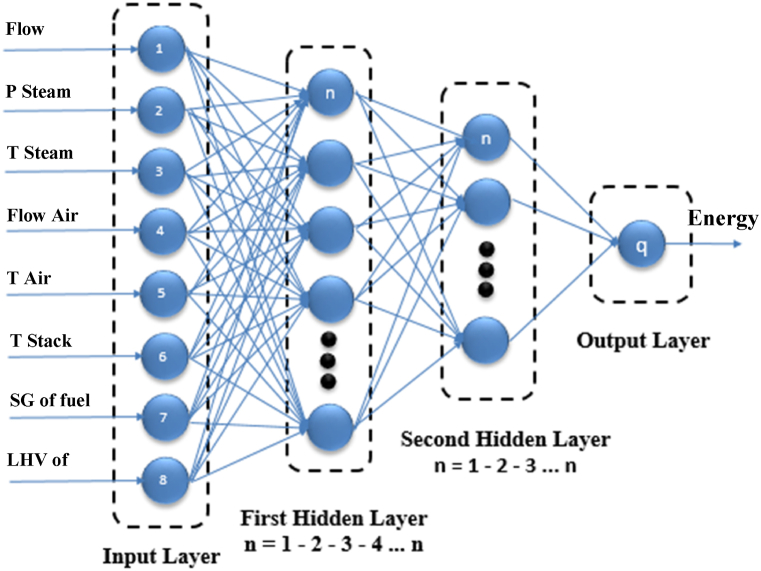
The structure of the MLP with related Inputs and outputs.

The MLP neural network's structure, which consists of an input layer, many hidden layers, and an output layer, is seen in Fig. 4. Reducing the mean square of the general error is the goal of the training method, which is split into two sections: forward pass and backward pass. In the forward pass, the network output is measured after the input vector is added, and in the backward pass, the error is computed as the discrepancy between the network output and the experimental data. First, this method makes the assumption that the weights and biases' values are correlated with the distribution's unknown variance. The following is one way to generate the MLP neural network's output. Where the bias weight for neuron j in the layer is denoted by j_kβ_, and the contribution of neuron j from layer k by y_jk_. Fk are nonlinear activation transfer functions, which might be identity, binary phase function, binary sigmoid, bipolar sigmoid, Gaussian, or linear functions. W_ijk_ are connection weights that are randomly selected at the start of the network training process [15].

The radial basis function (RBF) network, a kind of feedforward network with a single hidden layer, was initially presented by Brodhead and Lowe in 1998. Despite the similarities between the RBF and MLP approaches, the notion of hidden single-layer neurons differs greatly. The single hidden layer stores and passes the input layer data to the Gaussian transfer function, making the data nonlinear. In the RBF neural network, the transfer functions between the single hidden layer and the output layer are linear, but the transfer functions between the input layer and the single hidden layer are nonlinear. The RBF output layer is shown as a linear combiner in Eq. (5) [16].

(5) $$f\left(x\right)=\sum_{i=1}^{N}w_{ij}\;G\left(\|x-c_{i}\|*b\right)$$

where x stands for the input variable, ci for the central points, b for the bias, w_ijk_ for the weight applied to each hidden neuron, and N for the number of training data sets. Eq. (6) uses a Gaussian function to extract the focused response from the concealed point [17]:

(6) $$G\left(\|x-c_{i}\|*b\right)=\exp\left[\left(-\frac{1}{2\sigma_{i}^{2}}\;{\left(\|x-c_{i}\|*b\right)}^{2}\right)\right]$$

Where σ_i_ is the expansion of the Gaussian function. Range || x-c_i_|| shows in the input space to which the RBF neuron must respond [18].

## 4 Algorithm

**Fig. 5:**
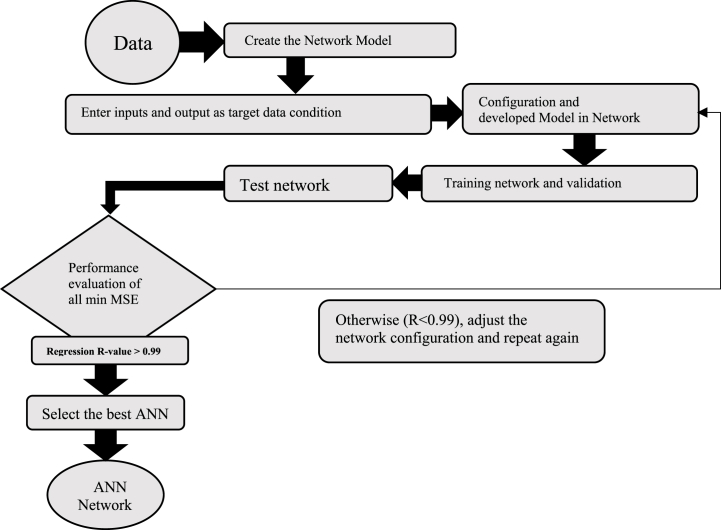
Workflow for model design.

Fig. 5 shows the work cycle for developing an ANN model. Data collection was done as the first step The following phase describes the ANN model as an input and output target, or target variables and parameters. A collection of data from the training phase is used to enhance the network validation data, and the test data is used to carry out the network validation procedure. The learning phase concludes when the generalizations are modified. In order to assess the correctness of the trained model, statistical parameters like mean square error (MSE) and R2 value have been used to compare the model's output with the data. The learning method begins with a high number of neurons in the single hidden layer and subsequently decreases this number as much as feasible since choosing neurons for the network is often a trial-and-error process. The algorithm training procedure concludes at the ideal error determined by data testing, and this neuron reduction is linked to decreasing the proportional error. The main goal of this research is to provide a general model for predicting equipment consumption in different operating conditions and choosing the most appropriate one to optimize fuel consumption [19].

## 5 Results and discussion

### 5.1 Aspen HYSYS results

**Fig. 6:**
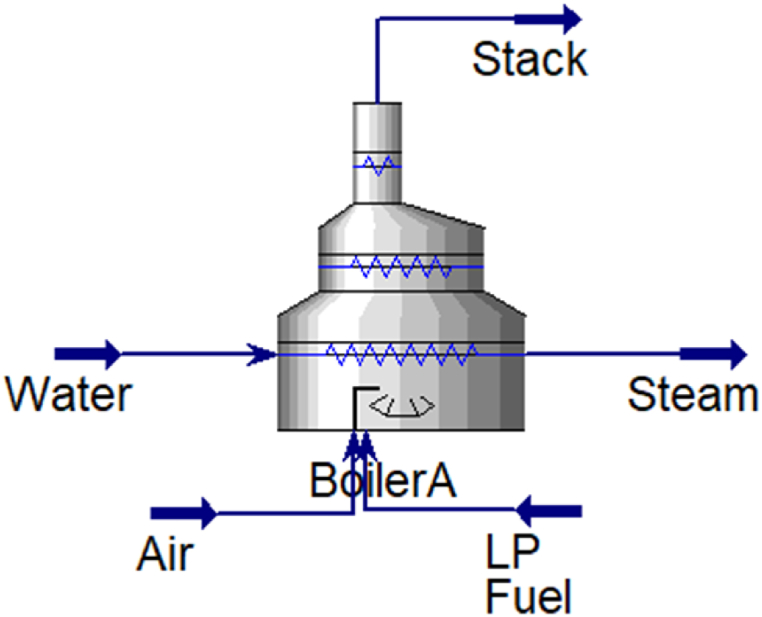
Boiler's PFD in Aspen HYSYS Version 14.

In Aspen HYSYS, the boiler can be simulated using either a heater or a furnace as a heat exchanger, each with specific conditions. In this study, the target boiler was simulated using a furnace in Aspen HYSYS, as the composition of the fuel percentage consumed is crucial and significantly affects fuel consumption. Fig. 6 illustrates the Process Flow Diagram (PFD) in Aspen HYSYS.

**Fig. 7:**
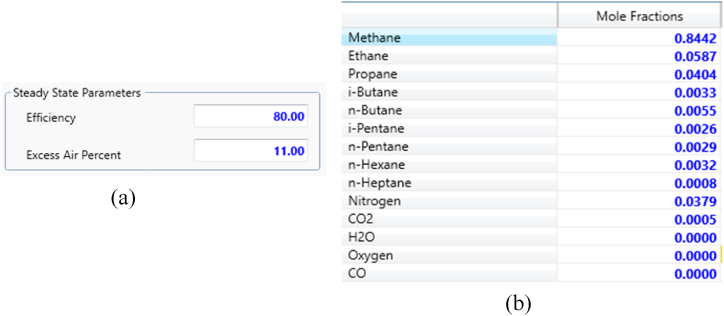
Input data for Aspen HYSYS Version 14.

To ensure the simulation closely reflects reality, the boiler efficiency was set at 80 %, with an excess air amount of 11 %. The fuel input composition for the equipment was accurately entered. The data provided by company staff, as shown in Fig. 7, were used for the simulation.

**Fig. 8:**
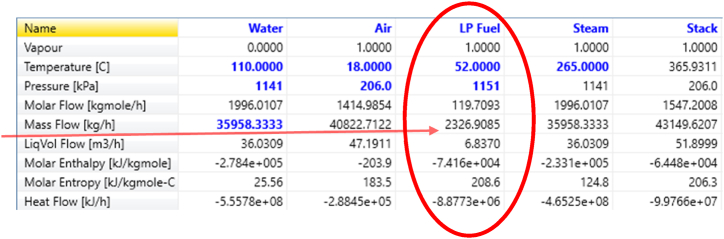
Output data from Aspen HYSYS Version 14.

The simulation was conducted using real values, and the results, shown in Fig. 8, represent the amount of fuel consumption.

**Fig. 9:**
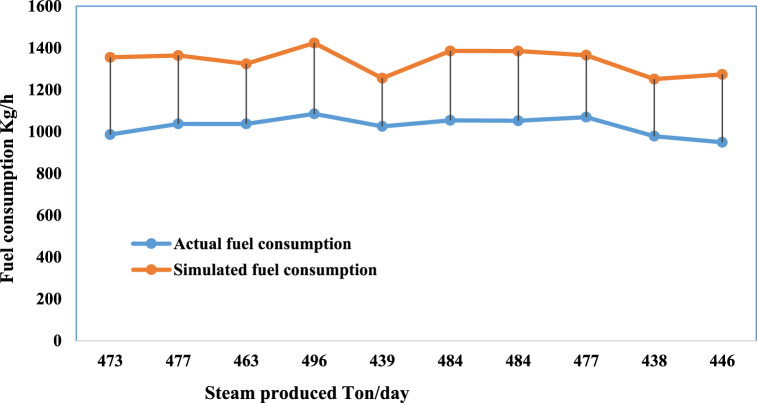
Comparison of Aspen HYSYS Version 14 response with experimental data.

After evaluating the results from Aspen HYSYS and assessing their accuracy, 10 operational points were simulated. Fig. 9 displays the comparison between simulated fuel consumption and actual fuel consumption.

Table 2 provides the data points shown in Fig. 9 along with the associated errors.

**Table 2:** Actual and simulated fuel consumption.

Actual fuel consumption	Simulated fuel consumption	Error %
986	1355.1	27.20
1037	1363.9	23.90
1037	1324.1	21.60
1085	1423.8	23.70
1025	1255.2	18.30
1054	1385.7	23.90
1052	1385.2	24.05
1069	1365.4	21.70
978.3	1251.3	21.80
949	1273.5	25.40

This research investigates boiler energy consumption using various methods to identify the most effective one. By comparing the error of each method, the method with the least error is deemed the most suitable. Although the error in Aspen HYSYS results is acceptable in the industry, it is not the sole criterion for selecting the best method.

### 5.2 RSM results

**Fig. 10:**
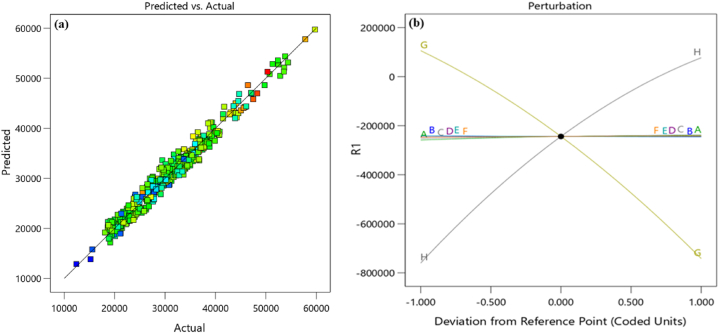
Images related to (a) the comparison of actual and predicted data and (b) the importance of input parameters.

For the RSM model, after entering the data and removing outliers, a quadratic model was proposed and selected. The predicted R^2^ of 0.9647 is reasonably close to the adjusted R^2^ of 0.9712, indicating a difference of less than 0.2. Adequate precision measures the signal-to-noise ratio, with a desirable ratio greater than 4. The obtained ratio of 104,616 signifies sufficient signal strength. Thus, the chosen model is deemed appropriate for exploring the design space. As illustrated in Fig. 10 (a), the results obtained from the RSM for predicted and actual values demonstrate high accuracy (R^2^ = 0.9741). Fig. 10 (b) highlights the significance of the input parameters on the response, clearly indicating that parameters G and H are highly influential in determining the response. Overall, the RSM results indicate that the model is effective for energy performance analysis, providing accurate and analyzable results [20].

### 5.3 MLP results

#### 5.3.1 Statistical results from MLP

**Fig. 11:**
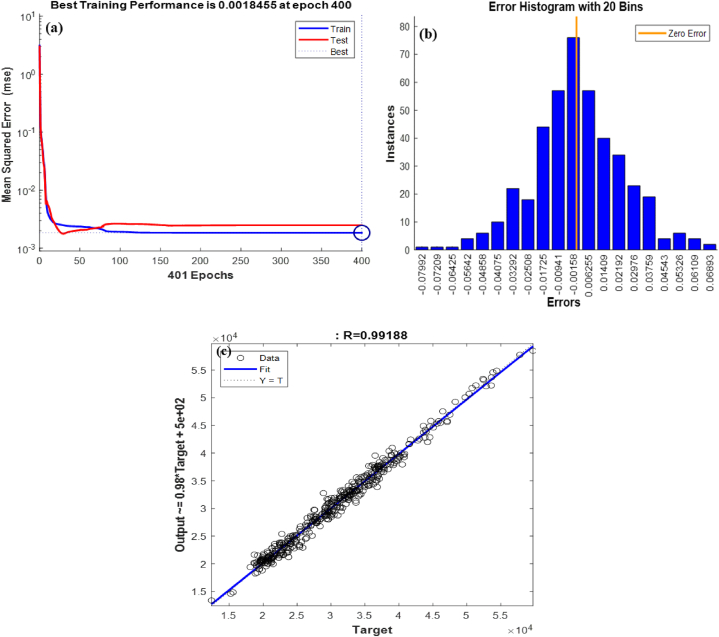
Data analysis by MLP (a) best training performance (b) error histogram (c) R^2^.

The MLP model comprises an input layer for data (e.g., flow, temperature, pressure) and an output layer for target data (energy consumption). The network has 8 inputs and 1 output, with two hidden layers: the first with 4 neurons and the second with 5 neurons. This configuration offers reasonable accuracy. Fig. 11(a–c) summarizes the results from the MLP, reporting a higher R^2^ value than the RSM (R^2^ = 0.99188). The normalized, predicted, and experimental results for boiler energy consumption demonstrate good agreement between the predicted models and actual data.

#### 5.3.2 Three-dimensional MLP diagram

##### 5.3.2.1 Interaction between the generated steam flow and reaction

**Fig. 12:**
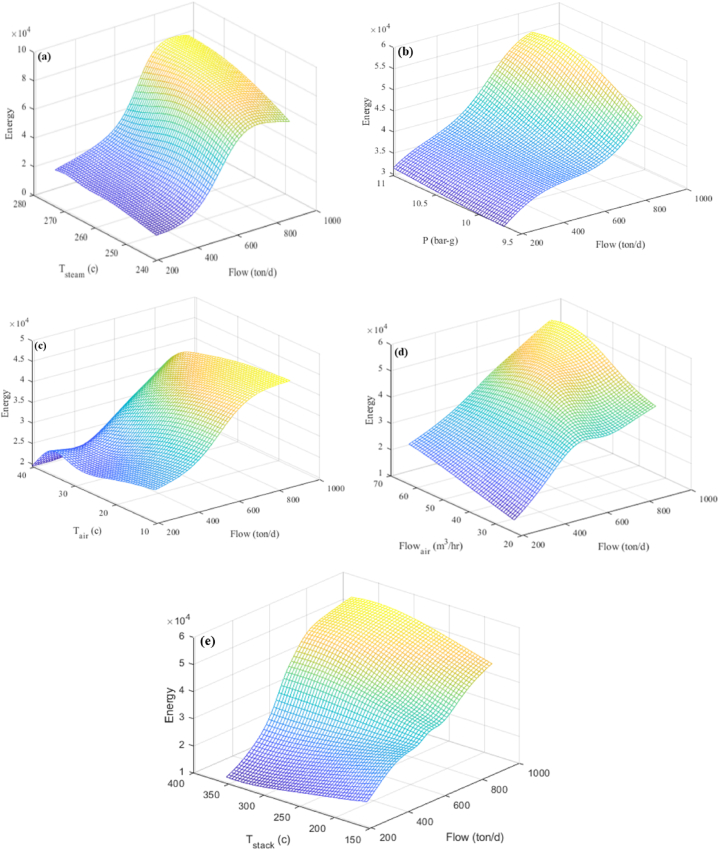
Three-dimensional diagram of produced steam flow and (a) steam temperature (b) steam pressure (c) air temperature entering the combustion stack (d) air flow entering the combustion stack (e) temperature of exhaust gases from the stack.

The 3D images created by MLP that give a summary of how input elements interact with the response and how each affects it are shown in Fig. 12(a–e). Fig. 12(a) displays data on steam flow varying from 530 to 550 tons per day, with temperatures between 240 and 270 °C and pressures about 10.3 barg. Steam flow data ranging from 600 to 800 tons/day with pressures between 10 and 11 barg and temperatures of 265.5 °C are shown in Fig. 12(b) as a consequence of burning 43,000 to 46,000 m³ of fuel with 42.84 m³/h of air at 25.5 °C. This is produced by mixing 56,000 to 66,000 m³ of gasoline with 42.84 m³/h of air at 25.5 °C, resulting in an exhaust gas temperature of 253.2 °C. In Fig. 12(d), data on steam flow from 480 to 600 tons per day at 256.5 °C and 10.3 barg of pressure are shown. This is produced by mixing 35,000 to 37,000 m³ of gasoline with 42.84 m³/h of air that is between 10 and 30 °C. Fig. 12(e) shows the steam flow data from 35,000 to 37,000 m³ of fuel with 42.84 m³/h of air at 25.5 °C, with temperatures of 256.5 °C and pressures varying from 10.3 barg.

##### 5.3.2.2 Interaction between pressure and reaction

**Fig. 13:**
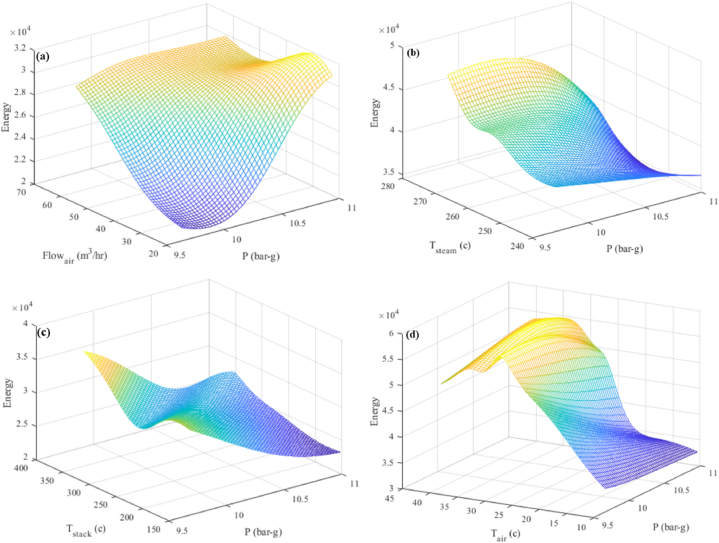
Three-dimensional diagram of produced steam pressure and (a) the air flow entering the combustion stack (b) steam temperature (c) the temperature of the air entering the combustion stack (d) the temperature of the exhaust gases from the chimney.

The pressure-response interaction graphs and their comparison with other parameters are displayed in Fig. 13(a–d). 525.5 tons of steam per day, with a temperature of 256.5 °C and a pressure of 9–11 barg, is generated by combining 26,000 to 28,000 m³ of fuel with 22–56 m³/h of air at 25.5 °C (Fig. 13(a)). Fig. 13 (b) shows that 525.5 tons of steam were generated every day using 41,000 to 43,000 m³ of fuel and 42.84 m³/h of air at 25.5 °C, with a pressure of 9–11 barg and a temperature of 250–270 °C. 525.5 tons of steam per day, with a temperature of 256.5 °C and a pressure of 9–11 barg, is generated by combining 32,000 to 30,000 m³ of fuel with 42.84 m³/h of air at 25.5 °C (Fig. 13(c)). 525.5 tons of steam per day, with a temperature of 256.5 °C and a pressure of 9–11 barg, is generated by combining 48,000 to 45,000 m³ of fuel with 42.8 m³/h of air at temperatures between 22 °C and 27 °C (Fig. 13(d)).

##### 5.3.2.3 Interaction between temperature of the produced steam and reaction

**Fig. 14:**
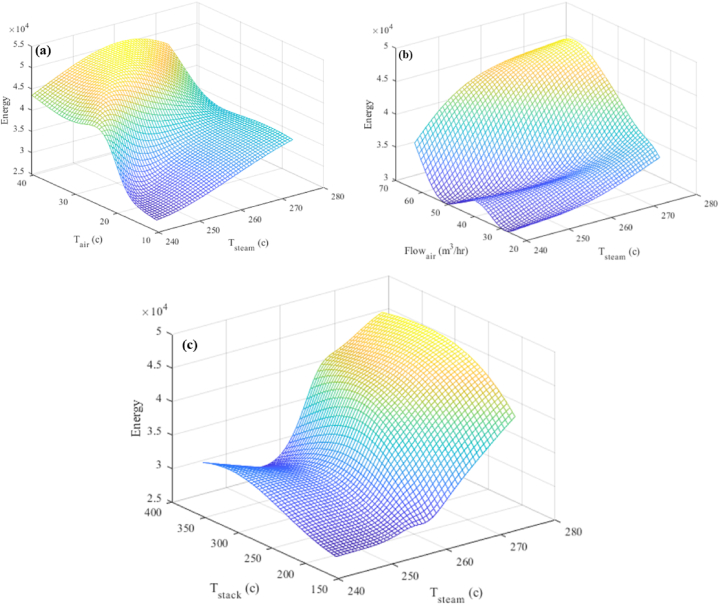
Three-dimensional diagram of the temperature of the produced steam and (a) the temperature of the air entering the combustion stack (b) the air flow entering the combustion stack (c) the temperature of the exhaust gases from the Stack.

Fig. 14(a–c) in this section also shows the interaction effect on the answer. Fig. 14(a) shows that between 44,000 and 40,000 m³ of fuel and 42.84 m³/h of air at temperatures between 20 °C and 38 °C produced 525.5 tons of steam per day at a pressure of 10.3 barg and a temperature of 240–270 °C. 525.5 tons of steam per day, at a temperature of 240–260 °C and a pressure of 10.3 barg, are generated from 42,000 to 40,000 m³ of fuel and 35–65 m³/h of air at 25.5 °C, as shown in Fig. 14(b). The temperature of the exhaust gas is 253.2 °C. 525.5 tons of steam per day, at a temperature of 250–270 °C and a pressure of 10.3 barg, are generated from 42,000 to 40,000 m³ of fuel and 42.84 m³/h of air at 25.5 °C (Fig. 14(c)).

##### 5.3.2.4 Interaction between the airflow rate and reaction

**Fig. 15:**
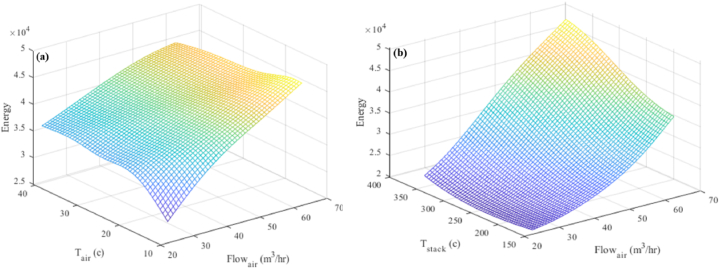
Three-dimensional diagram of the airflow entering the combustion stack and (a) the temperature of the air entering the combustion stack and (b) the temperature of the exhaust gases from the Stack.

Fig. 15(a and b) shows a three-dimensional depiction of the airflow rate and its impact on the response. 525.5 tons of steam per day, at a temperature of 256.5 °C and a pressure of 10.35 barg, are generated from 37,000 to 42,000 m³ of fuel and 30–50 m³/h of air at temperatures ranging from 10 °C to 40 °C (Fig. 15(a)). Fig. 15 (b) shows that 525.5 tons of steam were generated per day at a pressure of 10.3–256.5 barg using 43,000 to 36,000 m³ of fuel and 35–65 m³/h of air at 25.5 °C. The temperature of the exhaust gas is between 150 °C and 350 °C.

##### 5.3.2.5 Interaction between stack temperature and reaction

**Fig. 16:**
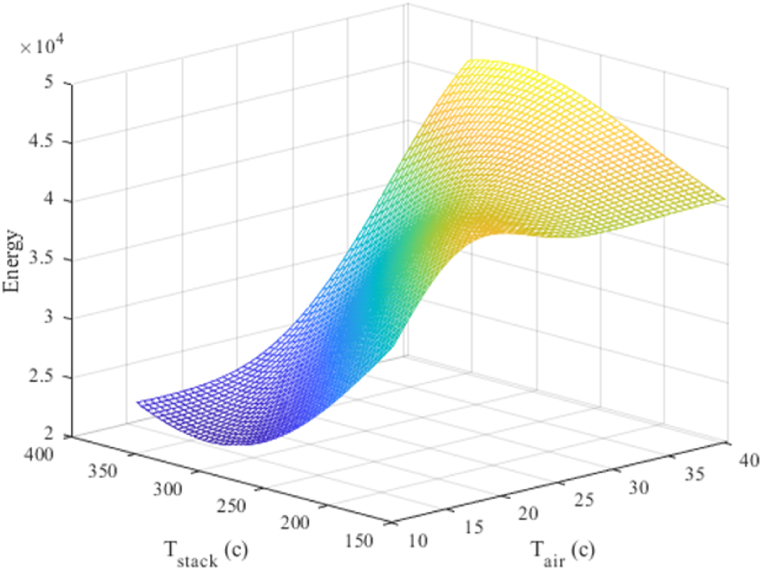
Three-dimensional diagram of the temperature of the air entering the combustion stack and the temperature of the exhaust gases from the stack.

525.5 tons of steam per day at a temperature of 256.5 °C and a pressure of 10.3 barg are generated from 34,000 to 39,000 m³ of fuel and 30–50 m³/h of air at temperatures ranging from 11 °C to 33 °C (Fig. 16). The temperature of the exhaust gas is between 150 °C and 350 °C.

### 5.4 RBF results

**Fig. 17:**
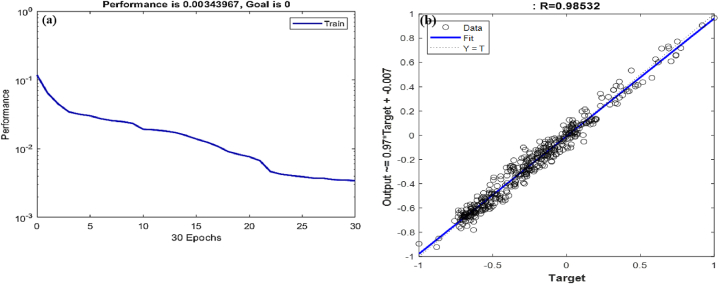
RBF performance with (a) epochs and (b) R^2^ of the RBF.

In contrast to the MLP, the RBF model is a form of neural network that has a single hidden layer. 30 neurons in the hidden layer were found to offer the best result after experimenting with different neuron counts. A strong match is shown by the RBF findings, which display an R2 value of 0.98532 (Fig. 17(b)). Furthermore, after 30 epochs, the performance value approached zero (0.00344), indicating a suitable and acceptable performance level (Fig. 17(a)).

## 6 Comparing results

In this section, the results obtained from the previous analyses are examined and compared. The analysis using RSM evaluates the data adequately; however, it is less accurate compared to neural network methods. It is evident from a comparison of Fig. 10, Fig. 11, Fig. 17 (b) that the R^2^ value for RSM is less than that of ANN, suggesting that ANN produces better outcomes than RSM. Specifically, the R^2^ value for RSM is 0.97, while ANN models yield R^2^ values of 0.98 for the RBF model and 0.99 for the MLP model. Fig. 11 (c) confirm that the MLP network performs the best in this research. Another comparison criterion is the performance value of each network. Fig. 11, Fig. 17 (a) show that the RBF network achieves a performance value of 0.0034, while the MLP network achieves a performance value of 0.0018. Since the goal is to minimize this value, a lower performance value indicates a more reliable model. Therefore, the MLP network is preferred based on this comparison. It appears that Aspen HYSYS does not accurately capture the actual energy consumption, leading to significant errors. Although Aspen HYSYS shows changes in fuel consumption that are somewhat proportional to actual changes, the predictions are not precise. Specifically, even with accurate fuel input percentages, Aspen HYSYS fails to accurately predict the lower heating value (LHV) of the fuel. Consequently, the MLP neural network model is recommended as the most suitable model for this research.

## 7 Conclusion

The best model for this study has been determined to be the MLP neural network model. Real operating points based on refinery data are contrasted with the operating points obtained from the network. Fuel consumption efficiency is expected to be improved by changes in operating parameters. Given that climate change is a significant worldwide issue, optimizing fuel usage is advantageous from an economic and environmental standpoint. The MLP results demonstrate that two important variables influencing fuel usage are the air entering the combustion stack and the steam pressure. Fuel use is greatly impacted by changes in these variables. Based on the findings, it is estimated that burning 26,000 m³ of fuel and 23 m³/h of air at 25.5 °C with an exhaust gas temperature of 253.2 °C might result in a production steam flow of 525.5 tons/day at a pressure of 10.5 barg and a temperature of 256.5 °C. A genuine operating point, on the other hand, reveals that the boiler generated 526 tons of steam per day at a temperature of 267 °C, a pressure of 10.3 barg, and a fuel consumption of 37,010 m³, using 52 m³/h of air at 35 °C. This implies that there was an excessive amount of air entering the combustion stack. Achieving this temperature is possible since the refinery is situated in a region of Iran where the air temperature is higher than 25 °C. An estimated 10,000 cubic meters of energy may be saved every hour by optimizing the operating point, preventing the emission of 27 tons of carbon dioxide. For comparison, this amount of CO₂ may be absorbed over 100 years by a 27-tree arboretum. From a financial standpoint, conserving 10,000 cubic meters of energy per day is equivalent to around $1,000 per day, $30,000 per month, and $360,000 annually. Future studies might examine other variables influencing boiler use, including the cooling fluid flow rate or the quality of the entering air and water.

## CRediT authorship contribution statement

**Erfan Gholamzadeh:** Writing – review & editing, Writing – original draft, Visualization, Methodology, Investigation, Formal analysis, Data curation, Conceptualization. **Ahad Ghaemi:** Writing – review & editing, Writing – original draft, Visualization, Project administration, Methodology, Investigation, Funding acquisition, Formal analysis, Data curation, Conceptualization. **Abolfazl Shokri:** Visualization, Validation, Software, Investigation, Data curation, Conceptualization. **Bahman Heydari:** Validation, Software, Methodology, Investigation, Conceptualization.

## Data availability statement

Data will be made available on request.

## Declaration of competing interest

The authors declare that they have no known competing financial interests or personal relationships that could have appeared to influence the work reported in this paper.

## References

[1] Nwankwo C.C.G. (2012). Process Plant Equipment.

[2] Shi Y., Zhong W., Chen X., Yu A.B., Li J. (2019). Combustion optimization of ultra supercritical boiler based on artificial intelligence. Energy.

[3] Smrekar J., Assadi M., Fast M., Kuštrin I., De S. (2009). Development of artificial neural network model for a coal-fired boiler using real plant data. Energy.

[4] Ganesan P., Rathna S.J., Saidur R. (Sep. 2021). Application of artificial neural network to map the performance characteristics of boiler using different algorithms. Int. J. Green Energy.

[5] Himeur Y. (2023). AI-big data analytics for building automation and management systems: a survey, actual challenges and future perspectives. Artif. Intell. Rev..

[6] Echi S., Bouabidi A., Driss Z., Abid M.S. (2019). CFD simulation and optimization of industrial boiler. Energy.

[7] Rashid H., Singh P. (2018). 2018 IEEE 4th International Conference on Collaboration and Internet Computing (CIC).

[8] Rashid H., Singh P., Stankovic V., Stankovic L. (2019). Can non-intrusive load monitoring be used for identifying an appliance's anomalous behaviour?. Appl. Energy.

[9] Shokri A., Ghaemi A. (2024). Developing artificial neural networks and response surface methodology for evaluating CO2 absorption into K2CO3/piperazine solution. Case Stud. Chem. Environ. Eng..

[10] Shokri A., Shahhosseini S., Bazyari A. (2024). Nanoporous Metatitanic acid on γ-Al2O3 aerogel for higher CO2 adsorption capacity and lower energy consumption. Sci. Rep..

[11] Zafari P., Ghaemi A. (2023). Modeling and optimization of CO2 capture into mixed MEA-PZ amine solutions using machine learning based on ANN and RSM models. Results Eng..

[12] Khoshraftar Z., Ghaemi A. (2023). Prediction of CO2 solubility in water at high pressure and temperature via deep learning and response surface methodology. Case Stud. Chem. Environ. Eng..

[13] Gil Chaves I., López J., Zapata J., Leguizamón Robayo A., Niño G. (2015).

[14] Chu J., Shieh S.-S., Jang S.-S., Chien C.-I., Wan H.-P., Ko H.-H. (2003). Constrained optimization of combustion in a simulated coal-fired boiler using artificial neural network model and information analysis. Fuel.

[15] Shokri A., Larki M.A., Ghaemi A. (Dec. 2024). Retrieval of carbon and inorganic phosphorus during hydrothermal carbonization: ANN and RSM modeling. Heliyon.

[16] Luo X.J., Oyedele L.O., Ajayi A.O., Akinade O.O. (2020). Comparative study of machine learning-based multi-objective prediction framework for multiple building energy loads. Sustain. Cities Soc..

[17] Strušnik D., Golob M., Avsec J. (2015). Artificial neural networking model for the prediction of high efficiency boiler steam generation and distribution. Simulat. Model. Pract. Theor..

[18] Abourayya A. (2021).

[19] Salim H., Sultan K.F., Jawad R. (2019). Comparison between PID and artificial neural networks to control of boiler for steam power plant. J. Eng. Sci..

[20] Shokri A., Kamran-Pirzaman A. (2024). 2024 9th International Conference on Technology and Energy Management (ICTEM).

